# The Polymorphism Analyses of Short Tandem Repeats as a Basis for Understanding the Genetic Characteristics of the Guanzhong Han Population

**DOI:** 10.1155/2021/8887244

**Published:** 2021-02-25

**Authors:** Shuyan Mei, Yanfang Liu, Congying Zhao, Hui Xu, Shuanglin Li, Bofeng Zhu

**Affiliations:** ^1^Multi-Omics Innovative Research Center of Forensic Identification; Department of Forensic Genetics, School of Forensic Medicine, Southern Medical University, Guangzhou 510515, China; ^2^Key Laboratory of Shaanxi Province for Craniofacial Precision Medicine Research, College of Stomatology, Xi'an Jiaotong University, Xi'an 710004, China; ^3^Clinical Research Center of Shaanxi Province for Dental and Maxillofacial Diseases, College of Stomatology, Xi'an Jiaotong University, Xi'an 710004, China

## Abstract

The short tandem repeat (STR) loci are polymorphic markers in the combined DNA index system (CODIS) and non-CODIS STR loci. Due to the highly polymorphic characteristic of STR loci, they are popular and widely used in forensic DNA typing laboratories. In this study, 22 STR loci (1 CODIS, 21 non-CODIS STR loci) and an Amelogenin locus were genotyped and analyzed in 590 unrelated individuals of the Guanzhong Han population. None of the 22 STR loci deviated from the Hardy–Weinberg equilibrium, and all the loci were in the linkage equilibrium state. We observed 247 alleles, and the corresponding allelic frequencies ranged from 0.0008 to 0.3695 in the Guanzhong Han population. The combined power of discrimination and the cumulative exclusion probability was 0.999 999 999 999 999 999 999 999 999 346 36 and 0.999 999 999 709 74, respectively. The results including Nei's *D*_*A*_ genetic distance, multidimensional scaling analysis, and principal component analysis showed that the Guanzhong Han population has closer genetic affinities with Northern Han, Chengdu Han, and Xinjiang Hui groups from China based on allelic frequencies of 15 overlapped STR loci from Guanzhong Han and 13 reference groups. The present results indicated that Microreader™ 23sp ID kit included highly polymorphic loci, and it could be well used for individual identification, paternity testing, and population genetics in the Guanzhong Han population.

## 1. Introduction

Short tandem repeats (STRs), as the molecular genetic marker of DNA length polymorphism, are widely spread in the human genome and often used to study population genetics, individual identification, and paternity testing by forensic researchers [1, 2]. The US Federal Bureau of Investigation selected the 13 highly polymorphic autosomal STR loci as the core loci of the combined DNA index system (CODIS) in 1991, which were called the CODIS STR loci. Since then, many commercial STR kits have been manufactured that contained these 13 CODIS STR loci, such as AmpFℓSTR Identifiler kit and PowerPlex® 16 System kit [3]. Researchers from different countries have conducted different population studies based on these kits for the human genetic analysis and individual identification in forensic practice [4–8]. In order to improving the power of identification and reducing the probability of mismatching, seven polymorphic STR loci have been added to 13 CODIS STR loci, turning it to 20 CODIS STR loci [9]. However, it did not meet the purpose of forensic researchers sometimes. Most of researchers turned their attentions to non-CODIS STR loci. Compared with the CODIS STR loci, the advantage of non-CODIS STR loci is that there are a large number of highly polymorphic loci to choose from for researchers. Therefore, the combined CODIS STR loci and non-CODIS STR loci can not only improve the efficiency of individual identification of the whole system but also improve the performance of paternity testing.

The Han nationality is the largest of 56 ethnic groups in China, accounting for 91.59% of the national population [3, 10, 11]. Han individuals are widely distributed in China with different cultural backgrounds and genetic characteristics. Previous researches reported that there were significant differences in allelic frequency distributions of some STR loci in Han populations in different regions [12–14]. Therefore, STR loci could be used to study population genetics of Han nationality. Han populations in different regions have different research values for researchers with different research purposes. It is not only of great significance to the forensic medical research, but also the effective expansion of population genetic data and clarified the genetic characteristics and genetic relationships among Han populations in different regions.

The Guanzhong region is located in the central part of Shaanxi province containing the five cities of Xi'an, Tongchuan, Baoji, Xianyang, and Weinan. It accounts for 55623 square kilometers with a permanent population of more than 23 million [15]. Xi'an, known as Chang'an in ancient times, is the birthplace of Chinese Silk Road and one of the four ancient capitals of the world. Today, the Guanzhong region is an important passage connecting the south and north regions of Shaanxi province [16]. And the Microreader™ 23sp ID system (Suzhou Microread Genetics, Suzhou, Jiangsu, China) has been proved to be a useful tool for forensic practice, including 21 non-CODIS STR loci, one CODIS STR locus, and one gender identification locus (Amelogenin) [17]. In this research, the Microreader™ 23sp ID system was used to study the genetic polymorphisms of 22 autosome STR loci for the Han population in the Guanzhong region, and the effectiveness of this system was further verified. At the same time, 13 groups were selected as reference groups in this study based on 15 shared STR loci with the purpose of understanding the genetic relationships among the groups from China, including Xinjiang Hui [18], Northern Han [10], Southern Han [19], Chengdu Han [20], Xinjiang Uygur [21], Xinjiang Kazakh [22], Xinjiang Mongolian [23], Hainan Li [24], Zhejiang She [25], Guangdong Han [26], Hainan Han [27], Qinghai Tibetan [28], and Changsha Hui groups [29].

## 2. Materials and Methods

### 2.1. Samples Collection

According to the research purpose, the blood samples of 590 healthy volunteers in the Guanzhong region were collected and made into dried blood spot specimens. Before blood samples were collected, 264 male and 326 female volunteers who claimed that they were unrelated at least three generations understood and signed the informed consents. This project had been supported from the human and ethical committees of Xi'an Jiaotong University and Southern Medical University before it started.

### 2.2. Multiple PCR and Genotyping

All samples were directly and simultaneously amplified with 22 STR loci and the Amelogenin gender locus using Microreader™ 23sp ID kit without the DNA extraction. Multiplex PCR with the 25 *μ*l volume was performed by the GeneAmp® PCR 9700 Thermal Cycler (Applied Biosystems, Foster City, CA, USA). Then, PCR products labeled with fluorescent dyes were size-separated, detected using ABI PRISM® 3130xL Genetic Analyzer (Applied Biosystems, Foster City, CA, USA), and the results were analyzed by GeneMapper® ID-X 1.3 software (Applied Biosystems, Foster City, CA, USA). All the conditions and reagents in PCR and capillary electrophoresis were the same as previously reported [17, 18].

### 2.3. Statistical Analyses

Allelic frequencies and forensic parameters including probability of exclusion (PE), polymorphism information content (PIC), matching probability (MP), power of discrimination (PD), and observed heterozygosity (Ho) were calculated by modified PowerState spreadsheet (version 1.2). And it was used to analyze *P* values of Hardy–Weinberg equilibrium (HWE) tests as well. According to specification, ARLEQUIN software (version 3.5) [30] was used to calculate the expected heterozygosity (He). Linkage disequilibrium (LD) analyses of all pairwise loci among 22 autosomal STRs were performed by ARLEQUIN software (version 3.5) and SHEsis online software [31] in this study. Based on the allelic frequencies of 15 overlapped STR loci of the Guanzhong Han population and other 13 reference groups, the locus-by-locus *P* values were calculated by ARLEQUIN software (version 3.5) using analysis of molecular variance (AMOVA) method, and the calculations of Nei's *D*_*A*_ genetic distances [32] were performed by DISPAN software. The ggplot2 package was used to draw a triangular heat map to show the values of Nei's *D*_*A*_ more intuitively by *R* statistical software (version 3.0.2). A Neighbor-Joining tree (NJ-tree) and a circular phylogenetic tree were constructed by MEGA version 3.697 [33] and iTOL online software (https://itol.embl.de/itol.cgi) based on the values of Nei's *D*_*A*_ distances with the purpose of illustrating the genetic relationships among 14 different groups. In addition, an unrooted tree was plotted by PHYLIP software (version 3.69) on the basis of the allelic frequencies of 15 same STR loci. The multidimensional scaling analysis (MDS) diagram was plotted using an algorithm in PASW statistics software (version 18). In addition, the principal component analysis (PCA) diagram was constructed by MVSP software (version 3.1) based on allelic frequencies of 15 shared STR loci.

## 3. Results

### 3.1. Forensic Efficacies of 22 STRs in the Guanzhong Han Population

All the 22 STR loci were successfully genotyped in 590 unrelated samples. The *P* values of 22 STR loci for the HWE tests in the Han population resided in the Guanzhong region were presented in Table S1. Only one locus (D6S477) deviated from HWE due to its *P* value with lower than the significance level of 0.05, while the others were higher than the significance level. *P* values of the LD tests were presented in Table S2, and the results showed that the *P* values of 25 pairs of 231 pairs in 22 STR loci were less than 0.05. The pairwise actual correlation coefficient (*r*^2^) values of 22 STR loci were less than 0.01, demonstrating that there were no strong relationships between pairwise loci (Figure S1). There were no significant deviations from HWE (*P* ≤ 0.05/22) and linkage equilibrium (*P* ≤ 0.05/231) after using Bonferroni correction, indicating that these loci were independent of each other and could be performed in subsequent calculations using the multiplication rule.

**Figure 1:**
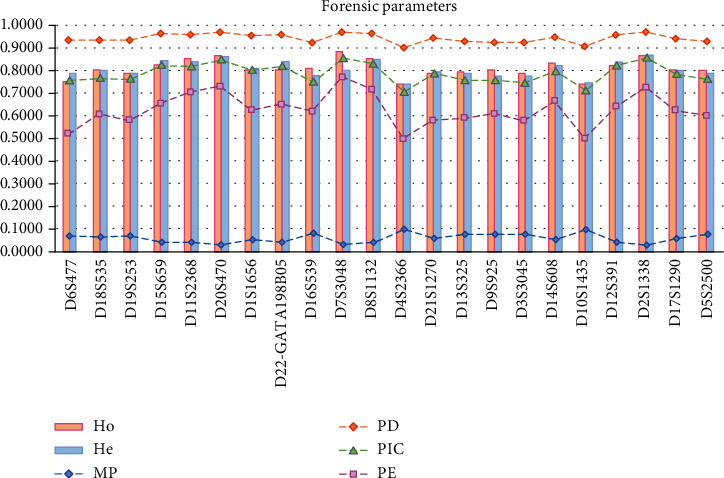
Forensic parameters of 22 STR loci in the Han population which dwelt in the Guanzhong region of Shaanxi province. The abscissa was the names of those loci, the ordinate was the values of forensic parameters (from 0 to 1), and the bottom was the names of the forensic parameters.

Allelic frequencies and forensic parameters of 22 STR loci were counted by Modified PowerState version 1.2 spreadsheet and shown in Table 1 and Figure 1, respectively. A total of 247 alleles were detected in this study, and the corresponding allelic frequencies ranged from 0.0008 to 0.3695. In the variations of 22 STR loci, the D4S2366 locus was the lowest allelic variations with 7 alleles, whereas the D1S1656 possessed the highest variations with 16 alleles. In this study, PIC values spanned from 0.7086 (D4S2366) to 0.8566 (D7S3048), indicating that all the 22 STR loci could provide highly reliable genetic information for the Guanzhong Han population. The Ho and He values ranged from 0.7407 (D10S1435) to 0.8881 (D7S3048), and 0.7474 (D4S2366) to 0.8711 (D7S3048), respectively. The comparisons of average PIC and Ho values in the 15 overlapped STRs between the Guanzhong Han population and 13 reference groups in China revealed that these STRs were highly genetic polymorphisms (Figure S2). The MP possessed the minimum value of 0.0319 (D2S1338) and the maximum value of 0.1004 (D4S2366). The PD values were in the range from 0.8996 (D4S2366) to 0.9681 (D2S1338). The minimum value of PE was 0.4940 (D10S1435), and the maximum PE value was 0.7713 (D7S3048). The further step was to calculate the combined performance of 22 STR loci, referring to the values of the combined power of discrimination (CPD) and the cumulative exclusion probability (CPE) that were 0.999 999 999 999 999 999 999 999 999 346 36 and 0.999 999 999 709 74, respectively. Furthermore, we compared the forensic parameters of this system with these of other commercial kits in the Shaanxi Han population previously reported, and the result in Figure S3 showed that polymorphisms of the 22 STR loci were relatively high in the Shaanxi Han population, followed by the Huaxia Platinum System [34], and the AGCU 21+1 STR system [15].

### 3.2. Analyses of Genetic Differences among 14 Groups

**Figure 2:**
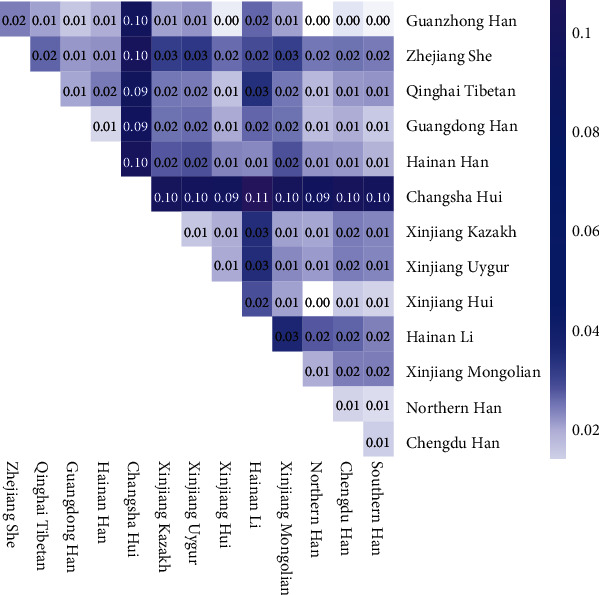
The heat map showing the genetic relationships among 14 groups based on Nei's *D*_*A*_ distances. The color ranged from white to blue to purple indicated the Nei's *D*_*A*_ distances from minimum to medium to maximum. The corresponding Nei's *D*_*A*_ values were displayed in each square grid (reserved two decimal fractions).

The locus-by-locus *P* values of the 15 same loci were calculated using AMOVA method and presented in Table 2 to explore the differences between Guanzhong Han and 13 reference groups. After Bonferroni correction (0.05/105 = 0.0005), there were significant differences between Guanzhong Han and Xinjiang Uygur, Xinjiang Kazakh, Hainan Li, Zhejiang She, Hainan Han, Xinjiang Hui, Qinghai Tibetan, Xinjiang Mongolian, Southern Han, Northern Han, and Changsha Hui populations at 12, 11, 11, 11, 6, 3, 2, 2, 2, 1, and 1 STR loci, respectively. The Nei's *D*_*A*_ values of the pairwise groups were shown in Table S3, and the intuitive heat map was shown in Figure 2, in order to explore the genetic distances among 14 groups. In the triangular heat map, the gradient from white to blue to purple represented the *D*_*A*_ values from small to median to large. The Hainan Li and Changsha Hui showed the maximum Nei's *D*_*A*_ value (0.1136), while Xinjiang Hui and Northern Han showed the minimum Nei's *D*_*A*_ value (0.0029). As far as the Guanzhong Han population, it showed the longest genetic distance with the Changsha Hui group (*D*_*A*_ = 0.0950), on the contrary, it presented the shortest genetic distance with the Northern Han population (*D*_*A*_ = 0.0030).

### 3.3. Three Phylogenetic Trees and Multidimensional Scaling Analysis of 14 Different Groups

**Figure 3:**
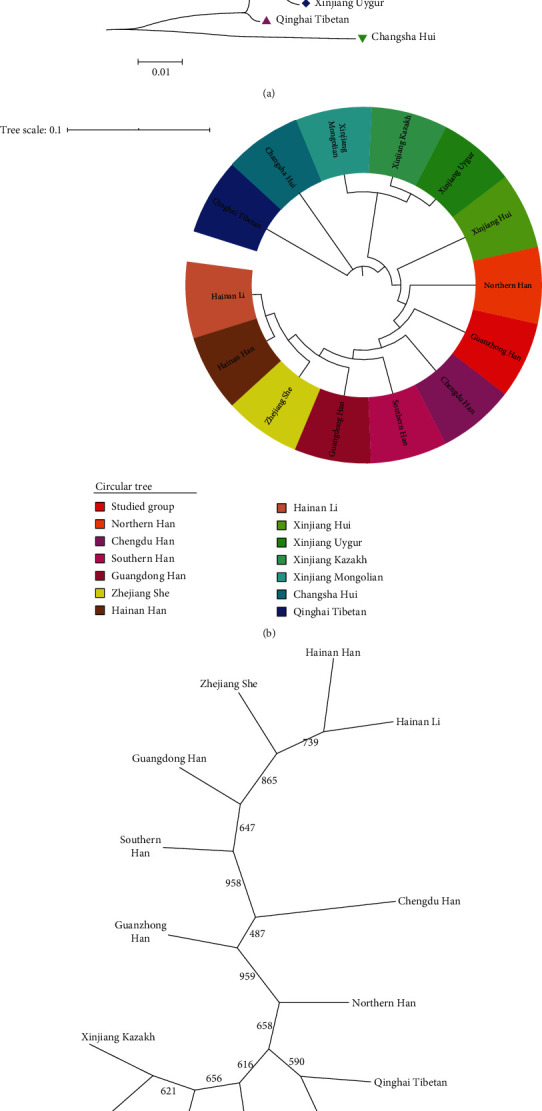
Phylogenetic trees among 14 different groups. The three trees intuitively reflected the genetic relationships among 14 groups. (a) Neighbor-Joining tree. Neighboring-Joining tree was produced by MEGA software. In the diagram, different shapes represented different populations or geographic locations, such as circle represented the Han population, and diamond represented the Xinjiang region. (b) Circular tree. The circular tree was drawn by iTOL online software. Different colors represented different populations (below). (c) Unrooted tree. The unrooted tree was constructed by PHYLIP v3.69 software.

The genetic relationships among different groups were intuitively exhibited in trees. The NJ-tree which was shown in Figure 3(a) was constructed by MEGA software based on Nei's *D*_*A*_ distances, and the circular phylogenetic tree presented in Figure 3(b) was drawn by iTOL software. An unrooted tree was constructed by PHYLIP Software v3.69 with the allelic frequencies of 15 overlapped STR loci and shown in Figure 3(c). NJ-tree has two major branches, one of which was the Changsha Hui and the other major branch contained three important sub-branches. The first minor branch was the Qinghai Tibetan group; the second one included Xinjiang Mongolian, Xinjiang Kazakh, and Xinjiang Uygur groups; however, the Han populations of six different regions in China, Xinjiang Hui, Hainan Li, and Zhejiang She groups formed the third subbranch. The two populations closest to the studied Guanzhong Han were the Northern Han and Chengdu Han populations. What is more, compared with other groups in Xinjiang region, Guanzhong Han had a smaller genetic distance to the Xinjiang Hui group. Similar results were observed in circular tree and unrooted tree.

**Figure 4:**
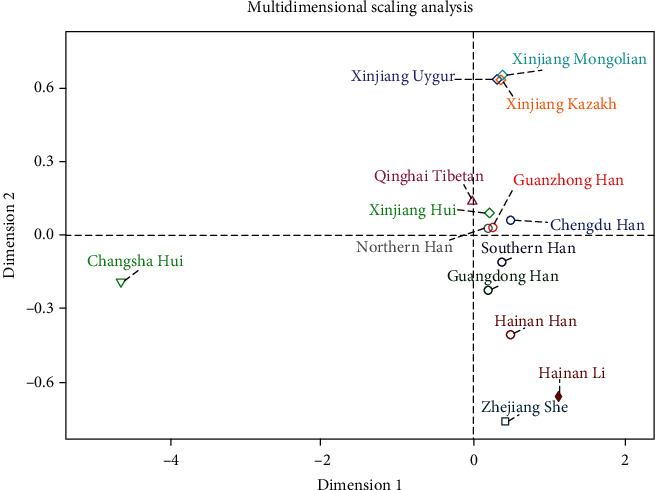
The MDS showing the genetic relationships among Guanzhong Han and 13 reference groups

Next, in order to further explore the genetic relationships of 14 populations, we constructed a MDS plot based on the pairwise Nei's *D*_*A*_ distances, which was presented in Figure 4. The Changsha Hui group was located in the lower left quadrant of the plot, which was far away from other groups. The Xinjiang Mongolian, Xinjiang Kazak, and Xinjiang Uygur groups were located at the upper right in the plot; and Zhejiang She, Hainan Li, and Hainan Han populations were in the lower right part of the plot, whereas the other seven groups (Qinghai Tibetan, Xinjiang Hui, Guanzhong Han, Chengdu Han, Northern Han, Southern Han, and Guangdong Han groups) were at the middle line in the plot, and the Northern Han located above the middle line was the closest population to the Guanzhong Han population. The MDS result showed that Guanzhong Han had relatively close genetic relationships with Northern Han, Chengdu Han, and Xinjiang Hui groups, which was consistent with the results of three trees.

### 3.4. Principal Component Analysis among the 14 Populations

**Figure 5:**
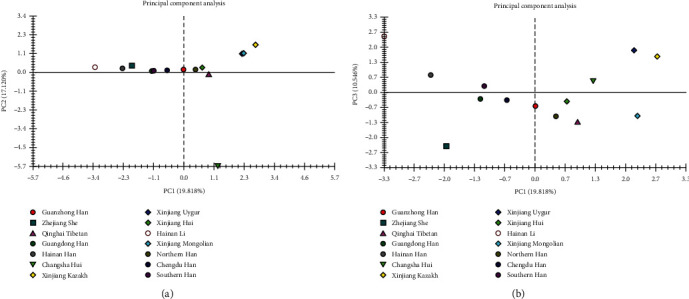
The principal component analysis plots showing genetic relationships between the Guanzhong Han population and 13 reference groups. The ratios of the first three principal components added up to 47.484%. Different colors and shapes represented different populations. (a) PC1 and PC2 scatter plot. (b) PC1 and PC3 scatter plot.

MVSP software was used to construct two PCA plots based on the allelic frequencies of 15 overlapped STR loci with the aim of demonstrating the genetic relationships among the 14 groups. The first, second, and third proportions of principal components accounted for a total of 47.484%. Different colors represented different populations in the two plots, and different shapes represented different populations or different regions; that is, the solid circle represented the Han population, the upward triangle represented the Tibetan group, the downward triangle represented the Hui group, the hollow circle represented the Li group, and the diamond represented the ethnic groups in the Xinjiang region. As shown in Figure 5(a), the Guanzhong Han population laid on the median line, with the nearest Northern Han, Chengdu Han, and Xinjiang Hui groups, but Hainan Li, Hainan Han, and Zhejiang She groups were at top left of the middle line in the graph which were far away from the Guanzhong Han population; and Xinjiang Kazak, Xinjiang Uygur, and Xinjiang Mongolian were on the right side of the figure. As shown in Figure 5(b), all populations were separated from each other, and the closest to the Guanzhong Han was the Northern Han population, followed by the Chengdu Han and the Xinjiang Hui groups. In general, the PCA result could well distinguish 14 groups from each other at the population level.

## 4. Discussion

Due to the complexity of population backgrounds and geographical features in China, population genetic researches of different ethnic groups in China have attracted much attentions in recent years [35]. Since ancient times, the Guanzhong region has always been a very important place in terms of the geographical location and natural resources. The core city of the Guanzhong region is Xi'an (Chang'an in ancient times) which is one of the important birthplaces of Chinese civilization, and it is the starting point of the overland Silk Road. What is more, at least a dozen dynasties established their capitals here in history. Therefore, as the center of ancient economic prosperity and cultural exchange, Guanzhong is one of the regions where researchers paied close attention to. Therefore, this is the reason why we chose the Guanzhong Han population as the object of this research.

The 22 STR loci conformed to the HWE in the Guanzhong Han population and all pairwise STR loci did not deviated from linkage equilibrium, which could be used for subsequent population genetic analyses. In addition, it would not be considered as linkage if the physical distance between two STR loci is more than 10 Mb in the human genome [36, 37]. The cumulative probability could be calculated because all the 22 STR loci were located on different chromosomes in this study which was exhibited non-linkage and the *P* values and *r*^2^ values of pairwise STRs in LD tests revealed that there was no LD in all pairwise loci at 22 STR loci.

A total of 247 alleles were found in the Guanzhong Han population. Among these 247 alleles, the minimum allelic frequency value was 0.0008, and the maximum allelic frequency was 0.3695. Generally speaking, a STR locus with PIC value greater than 0.5 indicates that it may provide high genetic information in the studied group. The Ho value of STR locus used in forensic genetic research should be more than 0.8 for forensic individual identification and paternity testing. In this study, the average PIC value was 0.7851, and the mean Ho value was 0.8088, which were similar to the values of other 13 reference groups based on 15 overlapped STR loci (Figure S2). In addition, we evaluated the efficacy of this kit by comparing the forensic parameters of 22 STR loci included in this kit in the Guanzhong Han population with that of these STR loci in two commercial kits (AGCU 21+1 STR system and Huaxia Platinum system) reported in the Shaanxi Han population. The average PIC, Ho, PD, and PE values of 22 STR loci in this study were higher than these of the other two kits, showing that the 22 STRs were highly polymorphic in the Guanzhong Han population, which could also be better used in the population genetic research. The CPD value was 0.999 999 999 999 999 999 999 999 999 346 36, which was much larger than that of the 13 CODIS STR loci (0.999 999 999 999 9851) [38], indicating that the 22 autosomal STR loci could be effectively used in individual identification and paternity testing in the Guanzhong Han population.

Allelic frequencies of the 15 STR loci were used to analyze the population differences between the Guanzhong Han and 13 reference groups published previously using AMOVA method in ARLEQUIN software, and the results showed that these loci with significant differences had population differentiation abilities between the Guanzhong Han population and the other 13 groups, which were suitable for the comparative study among populations [15]. Heat map based on Nei's *D*_*A*_ distances showed that the closest genetic distance to the Guanzhong Han population was the Northern Han (*D*_*A*_ = 0.0030) in this study, followed by the Southern Han (*D*_*A*_ = 0.0040) and the Xinjiang Hui (*D*_*A*_ = 0.0043). The three trees (rooted tree, unrooted tree, and circular tree) were constructed using three different softwares, and all the results indicated that the genetic distances between the Han populations in different regions were relatively close. MDS represented the spatial distribution of clusters in different populations and reflected the genetic differentiation pattern among populations to a certain extent. The genetic relationships between populations could be analyzed directly through the spatial distances between different populations in the MDS diagram. In the PCA diagram, the relationships between discrete points formed in the two-dimensional space on basis of allelic frequencies of 15 STR loci in every population were sufficient to reflect the genetic differentiations among populations. The results of MDS and two PCA plots were the similar as those of the phylogenetic trees in this research.

Previous research using HLA loci has explored the relationships between the Guanzhong Han population of Shaanxi province, Northern Han, and Southern Han populations and pointed out that these populations had closer genetic relationships [39]. Obviously, the result of the present study was consistent with the above result. The reasonable explanation of the close genetic relationships between the Guanzhong Han population and Han populations in different regions was that the Guanzhong region was an economic and cultural center in ancient times, and many immigrants chose to live here. Moreover, intermarriages between Han individuals in different regions of China have led to widespread gene exchange among different Han populations. There were close genetic relationships between Han populations and Xinjiang Hui group, but Han populations were far from the Changsha Hui group, which might be related to the complex origin of the Hui group. This phenomenon has been reported in previous literatures on the studies of Hui group [29, 39–42]. The present results on population genetics for the Guanzhong Han population provided valuable genetic information and reference data for the subsequent genetic relationship study of the Guanzhong Han population and also added the novel population data to the database of the Chinese Han nationality.

## 5. Conclusion

In this research, 22 autosomal STR loci were used to successfully typed and collected genetic information from 590 Han individuals dwelling in the Guanzhong region. The CPD and CPE values showed that the 22 STR loci could be applied to forensic individual identification and paternity testing for the Guanzhong Han population in daily forensic DNA cases. The results of the phylogenetic analyses indicated that there were relatively close genetic relationships between the Guanzhong Han population and the other Han populations in different regions of China and Xinjiang Hui group. In order to further reveal ancestral components for the Guanzhong Han population, more genetic markers will be used for detection and analysis in the future.

## Figures and Tables

**Table 1:** The allelic frequencies of 22 autosome STR loci in the Guanzhong Han population.

Alleles	D1S1656	Alleles	D2S1338	Alleles	D3S3045	Alleles	D4S2366
10	0.0008	16	0.0136	9	0.3390	9	0.3331
11	0.0746	17	0.0653	10	0.0305	10	0.0593
12	0.0398	18	0.1068	11	0.0373	11	0.3398
13	0.0831	19	0.1737	12	0.1254	12	0.1144
14	0.0856	20	0.1373	13	0.2305	13	0.0678
15	0.3102	21	0.0314	14	0.1737	14	0.0737
15.3	0.0008	22	0.0466	15	0.0627	15	0.0119
16	0.2237	23	0.1551	16	0.0008	Alleles	D9S925
16.3	0.0025	24	0.1831	Alleles	D8S1132	11	0.0008
17	0.0856	25	0.0644	16	0.0161	12	0.0017
17.3	0.0415	26	0.0212	17	0.0949	13	0.0144
18	0.0246	27	0.0008	18	0.2229	14	0.1449
18.3	0.0127	28	0.0008	19	0.1856	15	0.2186
19	0.0076	Alleles	D7S3048	20	0.1297	15.3	0.0008
19.3	0.0042	15.3	0.0008	21	0.1415	16	0.3051
20	0.0025	16	0.0008	22	0.1076	16.3	0.0076
Alleles	D6S477	17	0.0076	23	0.0814	17	0.2000
10	0.0085	18	0.0949	24	0.0136	17.3	0.0068
11	0.0034	19	0.0814	25	0.0068	18	0.0932
12	0.0593	20	0.1881	Alleles	D10S1435	19	0.0059
12.2	0.0008	21	0.1186	8	0.0203	Alleles	D12S391
13	0.2203	22	0.0805	9	0.0017	15	0.0136
14	0.1975	23	0.1381	10	0.0322	16	0.0076
15	0.2915	24	0.1729	11	0.1619	17	0.0797
16	0.1771	25	0.0924	12	0.3695	18	0.2254
17	0.0288	26	0.0212	13	0.2449	18.3	0.0034
18	0.0093	27	0.0025	14	0.1517	19	0.2076
19	0.0025	Alleles	D14S608	15	0.0144	20	0.1788
Alleles	D13S325	5	0.0017	16	0.0034	21	0.1178
15	0.0008	6	0.0822	Alleles	D16S539	22	0.0881
16	0.0008	7	0.2127	8	0.0093	23	0.0398
17	0.0076	8	0.0212	9	0.2661	24	0.0263
18	0.0542	9	0.0975	10	0.1119	25	0.0102
19	0.2390	10	0.2297	11	0.2669	26	0.0017
20	0.3042	11	0.2322	12	0.2161	Alleles	D18S535
21	0.1949	12	0.0924	13	0.1144	9	0.2127
22	0.1339	13	0.0280	14	0.0144	10	0.0492
23	0.0466	14	0.0017	15	0.0008	11	0.0280
24	0.0136	15	0.0008	Alleles	D21S1270	12	0.0949
25	0.0042	Alleles	D22-GATA198B05	9	0.0025	13	0.2127
Alleles	D15S659	14	0.0051	9.3	0.0025	14	0.2924
8	0.0051	15	0.0186	10	0.2941	15	0.1017
9	0.0017	16	0.0864	11	0.0610	16	0.0085
10	0.0169	17	0.1492	11.3	0.0008	Alleles	D19S253
11	0.1381	18	0.0669	12	0.0500	7	0.1610
12	0.2246	19	0.0958	12.3	0.0415	8	0.0373
13	0.1161	20	0.1017	13	0.1356	9	0.0085
14	0.0415	21	0.2856	13.3	0.0347	10	0.0195
15	0.1458	22	0.1568	14	0.2432	11	0.1441
16	0.1941	23	0.0297	14.3	0.0059	12	0.3356
17	0.0941	24	0.0034	15	0.1153	13	0.1975
18	0.0212	25	0.0008	16	0.0127	14	0.0839
19	0.0008	Alleles	D20S470	Alleles	D17S1290	15	0.0119
Alleles	D11S2368	6	0.0008	10	0.0347	16	0.0008
15	0.0042	9	0.0203	11	0.0415	Alleles	D5S2500
16	0.0364	10	0.1415	12	0.0169	9	0.0017
17	0.1500	11	0.0322	13	0.0093	10	0.0169
18	0.1034	12	0.0364	14	0.0220	11	0.2847
19	0.1551	13	0.1102	15	0.2331	12	0.1449
20	0.1636	14	0.1492	16	0.2992	13	0.0500
21	0.2500	15	0.1627	17	0.1746	14	0.1076
22	0.0915	16	0.1898	18	0.1025	15	0.2907
23	0.0347	16.2	0.0008	18.3	0.0051	16	0.0847
24	0.0085	17	0.1127	19	0.0398	17	0.0161
25	0.0025	18	0.0331	20	0.0153	18	0.0025
		19	0.0076	21	0.0042		
		20	0.0025	22	0.0017		

**(a):** 

Loci	Zhejiang She	Qinghai Tibetan	Guangdong Han	Hainan Han
D1S1656	***0.0000***	0.0059	0.1441	***0.0000***
D3S3045	***0.0000***	0.0296	0.0023	***0.0000***
D4S2366	0.0072	***0.0000***	0.2015	0.4501
D5S2500	***0.0000***	0.2302	0.1268	0.5336
D6S477	***0.0001***	0.1152	0.6364	***0.0000***
D7S3048	***0.0000***	0.0676	0.0152	0.0055
D8S1132	***0.0000***	0.1014	0.0175	0.0569
D10S1435	0.1692	0.2890	0.5016	0.0201
D11S2368	***0.0001***	0.4939	0.5137	0.3192
D13S325	***0.0000***	0.8291	0.1135	***0.0002***
D14S608	0.0404	0.0065	0.1068	0.1747
D15S659	***0.0000***	0.3444	0.2820	0.2530
D17S1290	0.0210	0.3354	0.0028	***0.0004***
D18S535	***0.0000***	***0.0001***	0.0182	***0.0000***
D22-GATA198B05	***0.0000***	0.3834	0.0927	0.0861

**(b):** 

Alleles	Changsha Hui	Xinjiang Kazakh	Xinjiang Uygur	Xinjiang Hui
D1S1656	***0.0000***	***0.0000***	***0.0000***	***0.0000***
D3S3045	0.6005	***0.0000***	***0.0000***	0.0326
D4S2366	0.7773	***0.0000***	***0.0000***	0.0316
D5S2500	0.0263	***0.0000***	***0.0000***	***0.0000***
D6S477	0.0330	***0.0000***	***0.0000***	***0.0000***
D7S3048	0.0932	0.0023	***0.0000***	0.4982
D8S1132	0.1248	***0.0000***	***0.0000***	0.0653
D10S1435	0.1201	***0.0000***	***0.0000***	0.9403
D11S2368	0.7482	0.2128	0.0006	0.0907
D13S325	0.4095	0.0005	0.0031	0.1652
D14S608	0.4958	***0.0000***	***0.0000***	0.0058
D15S659	0.7710	***0.0000***	***0.0000***	0.5340
D17S1290	0.4476	0.0015	***0.0000***	0.0215
D18S535	0.4349	***0.0000***	***0.0000***	0.0032
D22-GATA198B05	0.4875	***0.0000***	0.0005	0.8327

**(c):** 

Alleles	Hainan Li	Xinjiang Mongolian	Northern Han	Chengdu Han	Southern Han
D1S1656	***0.0003***	***0.0002***	***0.0000***	0.3634	***0.0000***
D3S3045	***0.0000***	0.0279	0.0895	0.4887	0.0031
D4S2366	***0.0000***	0.0025	0.0228	0.4494	0.1071
D5S2500	0.0123	0.0484	0.0005	0.2666	0.0210
D6S477	***0.0000***	***0.0000***	0.9002	0.8406	0.7620
D7S3048	***0.0000***	0.1147	0.0338	0.2094	0.1562
D8S1132	***0.0000***	0.0037	0.1978	0.6050	0.0277
D10S1435	0.0195	0.2083	0.0489	0.7157	0.0884
D11S2368	0.0235	0.6954	0.3865	0.0897	0.6508
D13S325	***0.0000***	0.0010	0.9197	0.9440	0.0426
D14S608	0.0219	0.0016	0.0248	0.8187	***0.0000***
D15S659	***0.0003***	0.6954	0.0612	0.2491	0.2474
D17S1290	***0.0000***	0.0999	0.0082	0.2270	0.0073
D18S535	***0.0000***	0.4713	0.0830	0.7735	0.7016
D22-GATA198B05	***0.0000***	0.0142	0.7097	0.9892	0.4431

The values in bold italics indicated that there were significant differences between the Guanzhong Han group and the comparison groups after Bonforroni correction (*P* ≤ 0.05/105 ≤ 0.0005).

## Data Availability

Data supporting the results of this study are available from the corresponding author upon request.

## Supplementary Materials

Supplementary 1 Supplementary Figure 1: actual correlation coefficient (*r*^2^) values of linkage disequilibrium analyses between pairwise STR loci were performed by SHEsis online tool in the Guanzhong Han population. The data in each rhomboid represented the *r*^2^ values magnified 100 times. The *r*^2^ values with less than 0.01 indicated that there were no strong relationships between corresponding two loci. Supplementary Figure 2: the comparisons of PIC and Ho values among different groups using the 15 overlapped STR loci. The blue represented mean values of PIC, and the yellow represented the mean values of Ho in different groups. Supplementary Figure 3: scatter plot for the comparisons of four forensic parameters between three different commercial kits of the Han population in Shaanxi province. Blue was the AGCU 21+1 STR system, orange represented the Microreader^TM^ 23sp ID kit used in this study, and gray meant the Huaxia Platinum System.

Supplementary 2 Supplementary Table 1: the *P* values of 22 STR loci of the Hardy–Weinberg equilibrium (HWE) tests in the Guanzhong Han population.

Supplementary 3 Supplementary Table 2: the *P* values of 22 STR loci in the linkage disequilibrium (LD) tests of the Guanzhong Han population. The bold values in this table indicated that the *P* values for the pairwise STR loci were less than the significance level 0.05.

Supplementary 4 Supplementary Table 3: the values of Nei's *D*_*A*_ distances of pairwise groups in 14 groups. The black italic boldface indicated the maximum (0.0950) and minimum (0.0030) genetic distances between the Guanzhong Han population and the 13 reference groups. The values of black upright and bold indicated the maximum (Changsha Hui and Hainan Li, 0.1136) and the minimum (Xinjiang Hui and Northern Han, 0.0029) of genetic distances between pairwise groups in the 14 groups.
