# Supplementary material for: The Polymorphism Analyses of Short Tandem Repeats as a Basis for Understanding the Genetic Characteristics of the Guanzhong Han Population
Source: Biomed Res Int. 2021 Feb 25;2021:8887244. doi: 10.1155/2021/8887244 (PMC7936557; doi:10.1155/2021/8887244)

**Supplementary Figure 1** Actual correlation coefficient (*r^2^*) values of Linkage disequilibrium analyses between pairwise STR loci were performed by SHEsis online tool in the Guanzhong Han population. The data in each rhomboid represented the *r^2^* values magnified 100 times. The *r^2^* values with less than 0.01 indicated that there were no strong relationships between corresponding two loci.


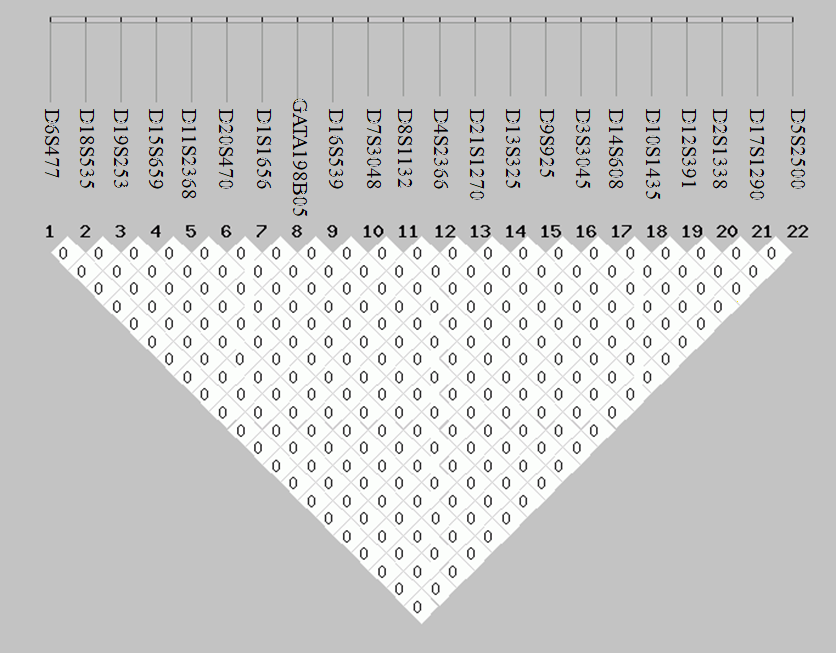


**Supplementary Figure 2** The comparisons of PIC and Ho values among different groups using the overlapping 15 STR loci. The blue represented meant values of PIC, and the yellow represented the mean values of Ho in different groups.

**Supplementary Figure 3** Scatter plot for the comparison of four forensic parameters between three different commercial kits of Han population in Shaanxi province. Blue was the AGCU 21+1 STR system, orange represented the Microreader^TM^ 23sp ID kit used in this study, and gray meant Huaxia Platinum System.


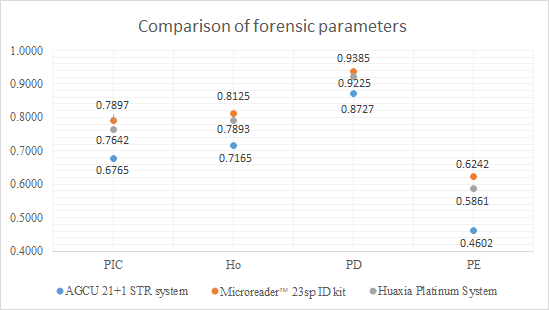

Supplement: Supplementary 1 — Supplementary Figure 1: actual correlation coefficient (r2) values of linkage disequilibrium analyses between pairwise STR loci were performed by SHEsis online tool in the Guanzhong Han population. The data in each rhomboid represented the r2 values magnified 100 times. The r2 values with less than 0.01 indicated that there were no strong relationships between corresponding two loci. Supplementary Figure 2: the comparisons of PIC and Ho values among different groups using the 15 overlapped STR loci. The blue represented mean values of PIC, and the yellow represented the mean values of Ho in different groups. Supplementary Figure 3: scatter plot for the comparisons of four forensic parameters between three different commercial kits of the Han population in Shaanxi province. Blue was the AGCU 21+1 STR system, orange represented the MicroreaderTM 23sp ID kit used in this study, and gray meant the Huaxia Platinum System. [file 8887244.f1.docx]
