# Supplementary material for: The Polymorphism Analyses of Short Tandem Repeats as a Basis for Understanding the Genetic Characteristics of the Guanzhong Han Population
Source: Biomed Res Int. 2021 Feb 25;2021:8887244. doi: 10.1155/2021/8887244 (PMC7936557; doi:10.1155/2021/8887244)
Supplement: Supplementary 2 — Supplementary Table 1: the P values of 22 STR loci of the Hardy–Weinberg equilibrium (HWE) tests in the Guanzhong Han population. [file 8887244.f2.docx]

**Supplementary Table 1** The *P* values of 22 STR loci of the Hardy–Weinberg equilibrium (HWE) tests in Guanzhong Han population

| Loci | *P* values | Loci | *P* values | Loci | *P* values |
| --- | --- | --- | --- | --- | --- |
| D1S1656 | 0.4721 | D9S925 | 0.4160 | D16S539 | 0.1522 |
| D2S1338 | 0.7475 | D10S1435 | 0.4789 | D17S1290 | 0.8367 |
| D3S3045 | 0.6520 | D11S2368 | 0.4615 | D18S535 | 0.9366 |
| D4S2366 | 0.7768 | D12S391 | 0.1378 | D19S253 | 0.7542 |
| D5S2500 | 0.6437 | D13S325 | 0.8338 | D20S470 | 0.9391 |
| D6S477 | 0.0229 | D14S608 | 0.5482 | D21S1270 | 0.0851 |
| D7S3048 | 0.2179 | D15S659 | 0.1690 | D22-GATA198B05 | 0.2938 |
| D8S1132 | 0.6193 |  |  |  |  |
