# Supplementary material for: The Polymorphism Analyses of Short Tandem Repeats as a Basis for Understanding the Genetic Characteristics of the Guanzhong Han Population
Source: Biomed Res Int. 2021 Feb 25;2021:8887244. doi: 10.1155/2021/8887244 (PMC7936557; doi:10.1155/2021/8887244)
Supplement: Supplementary 3 — Supplementary Table 2: the P values of 22 STR loci in the linkage disequilibrium (LD) tests of the Guanzhong Han population. The bold values in this table indicated that the P values for the pairwise STR loci were less than the significance level 0.05. [file 8887244.f3.docx]

**Supplementary Table 2** The *P* values of 22 STR loci in the linkage disequilibrium (LD) tests of Guanzhong Han population.

| Loci | D6S477 | D18S535 | D19S253 | D15S659 | D11S2368 | D20S470 |
| --- | --- | --- | --- | --- | --- | --- |
| D18S535 | **0.0229** |  |  |  |  |  |
| D19S253 | 0.5534 | 0.5968 |  |  |  |  |
| D15S659 | 0.5242 | 0.5748 | 0.6651 |  |  |  |
| D11S2368 | 0.6362 | 0.7582 | 0.8341 | 0.1928 |  |  |
| D20S470 | 0.5274 | 0.6681 | **0.0453** | 0.7033 | 0.6984 |  |
| D1S1656 | 0.2390 | 0.8888 | 0.2690 | 0.6089 | 0.9223 | 0.5913 |
| D22-GATA198B05 | 0.6115 | 0.5842 | **0.0131** | 0.6280 | 0.1382 | 0.4344 |
| D16S539 | 0.3848 | 0.3330 | 0.0785 | 0.4847 | 0.8938 | 0.1312 |
| D7S3048 | 0.9732 | 0.3336 | 0.2337 | 0.5494 | 0.3941 | 0.0167 |
| D8S1132 | 0.6090 | 0.6488 | 0.4641 | 0.7800 | 0.6776 | 0.2669 |
| D4S2366 | 0.5561 | **0.0033** | 0.0592 | **0.0401** | 0.1774 | **0.0336** |
| D21S1270 | 0.7429 | 0.0774 | **0.0141** | 0.4599 | 0.3908 | 0.8730 |
| D13S325 | 0.7664 | 0.1650 | 0.8380 | 0.0625 | 0.8492 | 0.3588 |
| D9S925 | **0.0269** | 0.5915 | 0.5966 | 0.2173 | 0.9885 | 0.0645 |
| D3S3045 | 0.1365 | 0.4806 | 0.5970 | 0.5736 | 0.1861 | **0.0354** |
| D14S608 | 0.3611 | 0.0906 | **0.0052** | 0.8849 | 0.5198 | 0.8362 |
| D10S1435 | 0.5138 | 0.9933 | 0.6963 | 0.2362 | 0.0742 | 0.4979 |
| D12S391 | 0.3800 | 0.4829 | 0.2155 | 0.2038 | 0.4343 | 0.1504 |
| D2S1338 | 0.4758 | 0.7912 | 0.6724 | 0.1238 | 0.0729 | 0.2200 |
| D17S1290 | 0.3897 | **0.0356** | 0.8077 | 0.0551 | 0.6293 | 0.5400 |
| D5S2500 | 0.1188 | 0.4657 | **0.0199** | 0.7792 | 0.9553 | 0.3049 |
|  |  |  |  |  |  |  |
| Loci | D1S1656 | D22-GATA198B05 | D16S539 | D7S3048 | D8S1132 | D4S2366 |
| D18S535 |  |  |  |  |  |  |
| D19S253 |  |  |  |  |  |  |
| D15S659 |  |  |  |  |  |  |
| D11S2368 |  |  |  |  |  |  |
| D20S470 |  |  |  |  |  |  |
| D1S1656 |  |  |  |  |  |  |
| D22-GATA198B05 | 0.8608 |  |  |  |  |  |
| D16S539 | 0.1337 | 0.6232 |  |  |  |  |
| D7S3048 | 0.1297 | **0.0090** | 0.5955 |  |  |  |
| D8S1132 | 0.4914 | 0.5298 | **0.0461** | 0.0682 |  |  |
| D4S2366 | 0.7724 | 0.8818 | 0.0891 | 0.9769 | 0.5499 |  |
| D21S1270 | 0.5274 | 0.1067 | **0.0060** | 0.2375 | 0.3219 | 0.0993 |
| D13S325 | 0.1641 | 0.1710 | 0.7270 | 0.8230 | **0.0474** | 0.2717 |
| D9S925 | 0.2400 | 0.1463 | 0.2420 | 0.7155 | 0.7133 | 0.4769 |
| D3S3045 | 0.1148 | 0.0914 | 0.5764 | 0.0971 | 0.1824 | 0.9008 |
| D14S608 | 0.2614 | 0.6486 | 0.2024 | 0.2480 | 0.3453 | 0.4043 |
| D10S1435 | 0.1079 | 0.6204 | 0.3068 | **0.0466** | 0.5802 | 0.0848 |
| D12S391 | 0.4829 | 0.4403 | 0.1752 | 0.9057 | **0.0185** | 0.7405 |
| D2S1338 | 0.0669 | 0.1525 | 0.3395 | 0.5020 | 0.4864 | 0.1987 |
| D17S1290 | 0.9557 | 0.4025 | 0.8164 | 0.7791 | 0.2253 | 0.1297 |
| D5S2500 | 0.1861 | 0.2698 | 0.4754 | 0.9377 | 0.3134 | 0.3320 |
|  |  |  |  |  |  |  |
| Loci | D21S1270 | D13S325 | D9S925 | D3S3045 | D14S608 | D10S1435 |
| D18S535 |  |  |  |  |  |  |
| D19S253 |  |  |  |  |  |  |
| D15S659 |  |  |  |  |  |  |
| D11S2368 |  |  |  |  |  |  |
| D20S470 |  |  |  |  |  |  |
| D1S1656 |  |  |  |  |  |  |
| D22-GATA198B05 | |  |  |  |  |  |
| D16S539 |  |  |  |  |  |  |
| D7S3048 |  |  |  |  |  |  |
| D8S1132 |  |  |  |  |  |  |
| D4S2366 |  |  |  |  |  |  |
| D21S1270 |  |  |  |  |  |  |
| D13S325 | 0.2666 |  |  |  |  |  |
| D9S925 | 0.3669 | 0.5466 |  |  |  |  |
| D3S3045 | **0.0050** | 0.8079 | 0.1994 |  |  |  |
| D14S608 | 0.4011 | 0.6801 | 0.3200 | 0.3814 |  |  |
| D10S1435 | **0.0174** | 0.2040 | 0.2164 | 0.9549 | 0.3423 |  |
| D12S391 | 0.4369 | 0.3451 | 0.4529 | 0.0852 | 0.2772 | 0.1120 |
| D2S1338 | 0.0602 | 0.4706 | 0.4037 | 0.2627 | 0.4041 | 0.9305 |
| D17S1290 | 0.1096 | 0.3511 | **0.0032** | 0.0784 | 0.3693 | **0.0052** |
| D5S2500 | 0.8889 | 0.2858 | 0.3420 | **0.0183** | 0.2436 | 0.0909 |
|  |  |  |  |  |  |  |
| Loci | D12S391 | D2S1338 | D17S1290 |  |  |  |
| D18S535 |  |  |  |  |  |  |
| D19S253 |  |  |  |  |  |  |
| D15S659 |  |  |  |  |  |  |
| D11S2368 |  |  |  |  |  |  |
| D20S470 |  |  |  |  |  |  |
| D1S1656 |  |  |  |  |  |  |
| D22-GATA198B05 | |  |  |  |  |  |
| D16S539 |  |  |  |  |  |  |
| D7S3048 |  |  |  |  |  |  |
| D8S1132 |  |  |  |  |  |  |
| D4S2366 |  |  |  |  |  |  |
| D21S1270 |  |  |  |  |  |  |
| D13S325 |  |  |  |  |  |  |
| D9S925 |  |  |  |  |  |  |
| D3S3045 |  |  |  |  |  |  |
| D14S608 |  |  |  |  |  |  |
| D10S1435 |  |  |  |  |  |  |
| D12S391 |  |  |  |  |  |  |
| D2S1338 | 0.4512 |  |  |  |  |  |
| D17S1290 | 0.0577 | **0.0356** |  |  |  |  |
| D5S2500 | **0.0004** | 0.6172 | 0.4876 |  |  |  |

The bold values in this table indicated that the *P* values for the pairwise STR loci were less than the significance level 005.
