# Supplementary material for: The Polymorphism Analyses of Short Tandem Repeats as a Basis for Understanding the Genetic Characteristics of the Guanzhong Han Population
Source: Biomed Res Int. 2021 Feb 25;2021:8887244. doi: 10.1155/2021/8887244 (PMC7936557; doi:10.1155/2021/8887244)
Supplement: Supplementary 4 — Supplementary Table 3: the values of Nei's DA distances of pairwise groups in 14 groups. The black italic boldface indicated the maximum (0.0950) and minimum (0.0030) genetic distances between the Guanzhong Han population and the 13 reference groups. The values of black upright and bold indicated the maximum (Changsha Hui and Hainan Li, 0.1136) and the minimum (Xinjiang Hui and Northern Han, 0.0029) of genetic distances between pairwise groups in the 14 groups. [file 8887244.f4.docx]

**Supplementary Table 3** The values of Nei’s *D_A_*-distances of pairwise groups in 14 groups.

| Nei’s *D_A_*-distance | Guanzhong Han | Zhejiang She | Qinghai Tibetan | Guangdong Han |
| --- | --- | --- | --- | --- |
| Zhejiang She | 0.0155 |  |  |  |
| Qinghai Tibetan | 0.0107 | 0.0197 |  |  |
| Guangdong Han | 0.0073 | 0.0121 | 0.0103 |  |
| Hainan Han | 0.0095 | 0.0132 | 0.0159 | 0.0056 |
| Changsha Hui | ***0.095*** | 0.1021 | 0.0917 | 0.0943 |
| Xinjiang Kazakh | 0.0109 | 0.0272 | 0.0167 | 0.0174 |
| Xinjiang Uygur | 0.0126 | 0.0288 | 0.0159 | 0.0183 |
| Xinjiang Hui | 0.0043 | 0.0182 | 0.0079 | 0.0099 |
| Hainan Li | 0.0192 | 0.0239 | 0.0307 | 0.0191 |
| Xinjiang Mongolian | 0.0111 | 0.027 | 0.0167 | 0.0176 |
| Northern Han | ***0.003*** | 0.0165 | 0.0089 | 0.0085 |
| Chengdu Han | 0.005 | 0.0176 | 0.0122 | 0.0098 |
| Southern Han | 0.004 | 0.0161 | 0.0125 | 0.0071 |

| Nei’s *D_A_*-distance | Hainan Han | Changsha Hui | Xinjiang Kazakh | Xinjiang Uygur |
| --- | --- | --- | --- | --- |
| Zhejiang She |  |  |  |  |
| Qinghai Tibetan |  |  |  |  |
| Guangdong Han |  |  |  |  |
| Hainan Han |  |  |  |  |
| Changsha Hui | 0.1008 |  |  |  |
| Xinjiang Kazakh | 0.0217 | 0.0995 |  |  |
| Xinjiang Uygur | 0.0229 | 0.0983 | 0.0075 |  |
| Xinjiang Hui | 0.0148 | 0.0947 | 0.0097 | 0.0099 |
| Hainan Li | 0.0141 | **0.1136** | 0.0316 | 0.032 |
| Xinjiang Mongolian | 0.0231 | 0.1003 | 0.0109 | 0.0138 |
| Northern Han | 0.0128 | 0.0941 | 0.0103 | 0.0119 |
| Chengdu Han | 0.0122 | 0.1009 | 0.0155 | 0.0159 |
| Southern Han | 0.0091 | 0.0978 | 0.0141 | 0.0145 |

| Nei’s *D_A_*-distance | Xinjiang Hui | Hainan Li | Xinjiang Mongolian | Northern Han | Chengdu Han |
| --- | --- | --- | --- | --- | --- |
| Zhejiang She |  |  |  |  |  |
| Qinghai Tibetan |  |  |  |  |  |
| Guangdong Han |  |  |  |  |  |
| Hainan Han |  |  |  |  |  |
| Changsha Hui |  |  |  |  |  |
| Xinjiang Kazakh |  |  |  |  |  |
| Xinjiang Uygur |  |  |  |  |  |
| Xinjiang Hui |  |  |  |  |  |
| Hainan Li | 0.0236 |  |  |  |  |
| Xinjiang Mongolian | 0.0107 | 0.0344 |  |  |  |
| Northern Han | **0.0029** | 0.0218 | 0.0098 |  |  |
| Chengdu Han | 0.0071 | 0.0196 | 0.0158 | 0.006 |  |
| Southern Han | 0.0063 | 0.0151 | 0.0155 | 0.0054 | 0.0067 |

The black italic boldface indicated the maximum (0.0950) and minimum (0.0030) genetic distances between the Guanzhong Han population and the 13 reference groups. The value of black upright and bold indicated the maximum (Changsha Hui and Hainan Li, 0.1136) and the minimum (Xinjiang Hui and Northern Han, 0.0029) of genetic distances between paired groups in the 14 groups.
